# Potential Damage to Modern Building Materials from 21st Century Air Pollution

**DOI:** 10.1100/tsw.2010.17

**Published:** 2010-01-21

**Authors:** Peter Brimblecombe, Carlota M. Grossi

**Affiliations:** School of Environmental Sciences, University of East Anglia, Norwich, UK

**Keywords:** multipollutant, climate change, aluminum, zinc, copper, plastic, paint, rubber

## Abstract

The evolution of damage to building materials has been estimated for the 21 century, with a particular focus on aluminum, zinc, copper, plastic, paint, and rubber in urban areas. We set idealized air pollution and climates to represent London and Prague across the period 1950–2100. Environmental parameters were used to estimate future recession, corrosion, and loss of properties through published damage or dose-response functions. The 21 century seems to provide a less aggressive environment for stone and metals than recent times. Improvements in air quality are the most relevant drivers for this amelioration. Changes in climate predicted for the 21 century do not alter this picture. On the other hand, polymeric materials, plastic, paint, and rubber might show slightly increased rates of degradation, to some extent the result of enhanced oxidant concentrations, but also the possibility of contributions from more solar radiation.

### INTRODUCTION

Traditional primary pollutants, such as sulfur dioxide, carbon monoxide, and smoke, declined markedly in the 20th century. This improvement in air quality tended to be experienced earlier in cities of Western Europe and North America, but came to be more widely felt by the end of the millennium, e.g., the major cities of China have recently witnessed such improvements. In parallel, there has been evidence that the declining concentrations of the more aggressive of these primary pollutants have caused less damage to urban buildings; they are, for example, less soot encrusted or corroded than in the past[1].

This is not to say that we are freed from concern over environmental threats to the state of urban fabric. It may well be placed at increased risk from automotive emissions[2], changes in climate[3] (in terms of salt damage[4], wind-driven rain, etc.), biological growth, alteration in the tone of façades or blackening patterns on buildings[5,6].

Some of the evidence for improvements in the condition of the urban fabric derives from a consideration of traditional building materials, such as stone, iron, copper, and glass, and their exposure to primary pollutants. The nature of the urban atmosphere has changed greatly, and the dominance of oxidants such as ozone or the presence of diesel soot may pose new hazards[1]. It is notable that these novel pollutants can be detrimental to modern paints and polymers[7], or cause discoloration of building façades[5]. This paper focuses on aluminum, zinc, copper, plastic, paint, and rubber and the potential hazards these materials face in a changing urban environment.

Here we examine the period 1950–2100. This starts prior to the 21st century to place our current era in the sharpest relief. The paper examines two idealized climates: (1) a maritime-influenced city like London, trying to capture its early regulation of air pollution, and (2) Prague, a continental city with a colder winter climate and a more central European ozone environment. We approximate the conditions in these cities, as we wish them to be seen as two different models for potential European air pollution and climate impact on building materials.

### AIR POLLUTION AND CLIMATE

An assessment of the effect of air pollution and climate first requires realistic estimates for meteorological variables and air pollutant concentrations (see Fig. 1) at our two idealized locations. Future climate was largely taken from output of the Hadley Model (HadCM3a2), while pollution estimates relied on assessments from likely policy changes as outlined below.

#### London

Sulfur dioxide and smoke were measured in London on a sporadic basis for much of the 20th century, but there are less than 50 years of measurements for air pollutants such as ozone and the nitrogen oxides. An overall trend for the years 1960–2000 is available for sulfur dioxide, nitrogen dioxide, and smoke[8], and ozone measurements from central London exist from 1973 (Fig. 1e). The ozone measurements (from stations such as Bloomsbury and Islington) give the sense of a relatively low ozone concentration in central London because of the nature of this secondary pollutant. In Greater London as a whole, the annual mean ozone increased about 50% over the period 1996–2006[9], but this sharp increase is not reflected very strongly in the time trends for central London. In the future, tighter regulation is likely to keep traditional pollutants, such as sulfur dioxide, low. Continued improvements come from European Union regulation and national initiatives. Additionally, London's former Mayor, Ken Livingstone, saw the environment as a key issue and was uncompromising in adopting the congestion charge, encouraging cleaner road vehicles and pressing for a Low Emission Zone. We assume in our calculations that London will continue to see an improved environment. London is presented as our low-ozone case such that its concentrations do not increase through the 21st century.

London's climate will change over the present century, with models suggesting a temperature rise around 4°C by 2080, and a precipitation increase in winter and decrease in summer under the a2 Emissions Scenario[10]. Future rainfall, temperature, relative humidity, and global radiation have been taken from the HadCM3a2 output[11], with slight adjustments for the effect of the urban heat island[12]. Such simple adjustments do not allow for more subtle changes in important parameters, such as surface wetness, which can affect the dissolution of acidic gases on material surfaces. Crude predictions of future surface wetness for Central England suggest a slight decrease over the coming century[13]. These results also suggest a decrease in the amount of surface wetness in the summer months. However, the lack of hourly resolution data means that it is not possible to establish whether the likely increases in night-time temperatures decrease nocturnal surface wetness even further. Values for rainfall pH begin at 4 and increase to 5 by the millennium, while chloride concentrations in precipitation have been set at 6 mg $l^{-1}$ for 1950, a period where high chloride coal was burned, declining to 3.5 mg $l^{-1}$ from 1990.

#### Prague

The available air pollution data for Prague[14,15] cover a shorter length than from London, but are nevertheless sufficient to establish our idealized trends. We have set both sulfur dioxide and particulate matter to decline in the late 20th century[15]. Nitrogen dioxide has been left constant over the 1950–2100

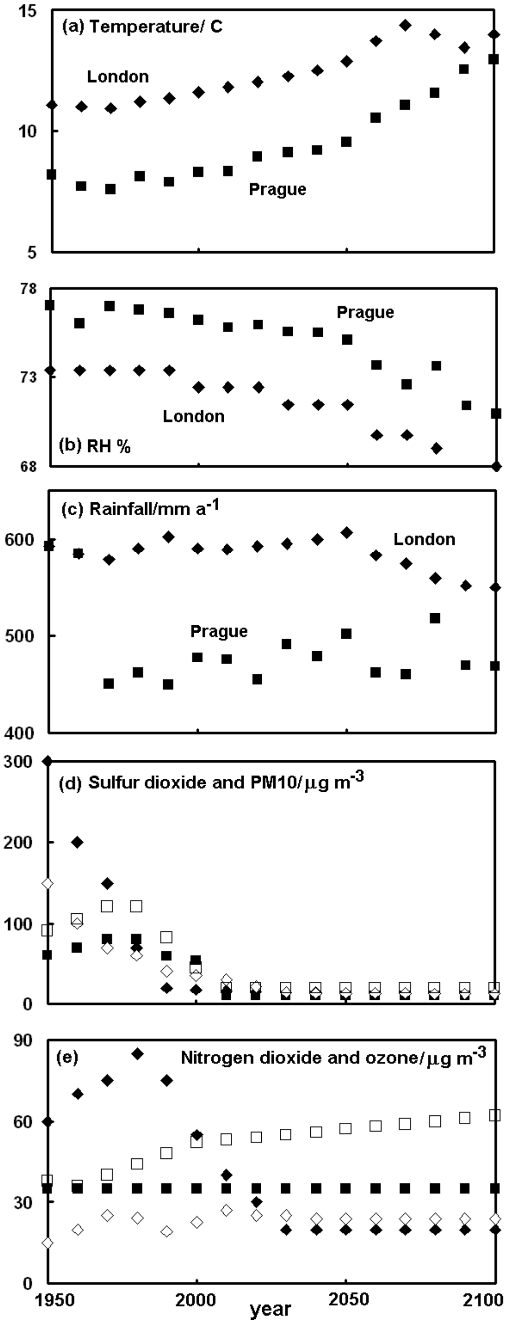

**FIGURE 1.** (a) Annual average temperatures adopted for London and Prague. (b) Annual average relative humidity adopted for London and Prague. (c) Annual average rainfall (i.e., precipitation less snowfall) adopted for London and Prague. (d) Average $SO_2$ (filled symbols) and $PM_{10}$ (unfilled symbols) concentrations for London (diamonds) and Prague (squares) used in the current study. Historic values of $SO_2$ and soot are available for London from 1950 and for Prague from 1981[15]. (e) Average $NO_2$ (filled symbols) and $O_3$ (unfilled symbols) concentrations used in the current study for London (diamonds) and Prague (squares). Historic values for London are available from the 1970s (http://www.airquality.co.uk/archive/index.php), while the measured data for Prague come from the 1990s.

period, but we have allowed a gradual ozone increase of close to 20% through the 21st century following some air pollution and climate change models[16]. The climate data are taken from a tuned set of output from HadCM3a2[17], which shows a substantial increase in temperature (Fig. 1a) and a decrease in relative humidity, but only the slightest upward trend in rainfall, mostly the result of a declining fraction of precipitation arriving as snow (Fig. 1b,c).

### Scenario Choice

Choosing Prague and London allows us to explore two quite different scenarios, particularly with respect to ozone, where its future is uncertain. Some predictions have foreseen an increase, assuming a changing climate, but no reduction in precursor emissions[16]. However, there are other scenarios where the European precursor emissions are likely to decrease[18], so a rise in ozone is not certain, but its control requires attention to precursor emissions at a regional scale[19].

## DAMAGE OR DOSE-RESPONSE FUNCTIONS

This work uses well-established damage or dose-response functions for a range of materials that characterize the modern built environment. We retain stone as a reference because so much work has been done on the response of carbonate materials. Damage to stone is often reported as recession in microns per annum[20], but other materials are expressed in different ways[21], so it is important to remain alert to this even though here we are interested in relative change in damage rate, rather than the absolute amounts.

### Stone

The Lipfert function[22] has been adopted here for recession of stone, but other functions, such as MULTI-ASSESS and ICP (derived from European Union MULTI-ASSESS[23] and United Nations ICP materials dose-response programs[21]) have been explored for London and Prague in earlier papers[17,24]:

$$-\mathrm{d}x/\mathrm{d}t = Lv \cdot Rn + 0.016[\mathrm{H}^+]Rn + 0.18(V_{dS}\,\mathrm{SO}_{2(g)} + V_{dN}\,\mathrm{HNO}_{3(g)}) \quad (1)$$

where $-\mathrm{d}x/\mathrm{d}t$ is the rate of recession as μm $a^{-1}$, $Rn$ is rainfall as m $a^{-1}$ (taken as rain, not precipitation, because snow is less effective at dissolution), $V_{dS}$ and $V_{dN}$ are deposition velocities (0.38 and 0.32 cm $sec^{-1}$[25]), $SO_{2(g)}$ and $HNO_{3(g)}$ are concentrations as μg $m^{-3}$, and $[H^+]$ is the concentration in rainwater as μmol $l^{-1}$. The term $Lv$ is the Lipfert value, which is typically set at 18.8 μm (recession) $m^{-1}$ (precipitation), but adjusted here for changing temperature and $CO_2$ partial pressure as in Bonazza et al.[20].

### Aluminum

In modern architecture, aluminum seems an iconic material. The mass loss of aluminum, $\Delta m_t$, over time periods is given by the ICP program as[21]:

$$\Delta m_t = 0.0021\mathrm{SO}_{2(g)}^{0.23} RH \exp(f_{\mathrm{Al}}) t^{1.2} + 0.023 Rn\, c_{\mathrm{Cl}}\, t \quad (2)$$

where $f_{\mathrm{Al}}$ is $0.031(T\text{-}10)$ when $T < 10^{\circ}$C and $-0.061(T\text{-}10)$ at $T > 10^{\circ}$C, $c_{\mathrm{Cl}}$ is the concentration of chloride ion (mg $l^{-1}$) in rainwater, and $t$ is time in years. Note the nonlinear dependence on some parameters.

Noninteger exponents for time, in particular, create a problem, so all of our calculations for metals (and later some plastics) represent only the first year of exposure and are thus initial rates.

### Zinc

Zinc is not often used in its pure form in buildings, although sculpture with the metal became popular in the 19th century[26]. Here, functions for mass loss in unsheltered ($\Delta m_t$) and sheltered ($\Delta m'_t$) areas are adopted from the ICP study[21]:

$$\Delta m_t = 1.35SO_{2(g)}^{0.22}\exp(0.018RH + f_{Zn})t^{0.85} + 0.029Rn[H^+]t \quad (3)$$

where $f_{Zn}$ is 0.062($T$-10) when $T < 10^oC$ and –0.021($T$-10) at $T > 10^oC$.

$$\Delta m'_t = 0.058SO_{2(g)}^{0.16}RH\exp(f'_{Zn})t^{0.49} \quad (4)$$

where $f'_{Zn}$ is 0.039($T$-10) when $T < 10^oC$ and –0.034($T$-10) at $T > 10^oC$.

The MULTI-ASSESS function[27] takes the form:

$$\Delta m_t = 1.82 + (1.71 + 0.471SO_{2(g)}^{0.22}\exp(0.018RH)\exp(f_{Zn}) + 0.041Rn[H^+] + 1.37HNO_{3(g)})t \quad (5)$$

### Copper

Copper is still used in construction, although it is also a traditional material. Here, we adopt the functions for mass loss in unsheltered ($\Delta m_t$) and sheltered ($\Delta m'_t$) areas from the ICP study[21]:

$$\Delta m_t = 0.0027SO_{2(g)}^{0.32}O_{3(g)}^{0.79}RH\exp(f_{Cu})t^{0.78} + 0.05Rn[H^+]t^{0.89} \quad (6)$$

where $f_{Cu}$ is 0.083($T$-10) when $T < 10^oC$ and –0.032($T$-10) at $T > 10^oC$.

$$\Delta m'_t = 0.00045SO_{2(g)}^{0.28}O_{3(g)}^{1.16}RH\exp(f'_{Cu})t^{0.78} \quad (7)$$

where $f'_{Cu}$ is 0.033($T$-10) when $T < 10^oC$ and –0.019($T$-10) at $T > 10^oC$.

We have chosen –0.019 rather than +0.019 of the original report[21], as it appears to be the correct constant. The MULTI-ASSESS function[27] takes the form:

$$\Delta m_t = 3.12 + (1.09 + 0.002SO_{2(g)}^{0.4}O_{3(g)}RH_{60}\exp(f_{Cu}) + 0.0878Rn[H^+])\,t \quad (8)$$

where $RH_{60} = RH - 60$ at $RH > 60$. Again, there is a nonlinear dependence on time and other parameters. The function from the National Material Exposure Program (NMEP)[28] is used as adopted by Brimblecombe and Grossi[24]:

$$-dm/dt = 8.92\,(2.2 + 0.025SO_{2(g)} + 37Rn)/4 \quad (9)$$

### Plastic

There is an enormous amount of literature on the degradation of polymer materials, but much is difficult to use in the manner adopted here. The amount of solar radiation and temperature is especially important. A simple equation based on the damage to polyethylene exposed in the open is:

$$IR_1 = 0.05*T \quad (10)$$

where $IR_1$ is a measurement of the IR spectra of polyethylene, related to the damage. However, this is dependent only on temperature and not pollutant concentrations. It is difficult to establish a clear relation between the parameter $IR_1$ exposure and damage measures, such as *breaking elongation*[21]. Time- and pollution-dependent functions are available for damage depth in polyurethane and color change in polyester[29,30]:

$$\Delta x = 0.5777 + 0.49G^{½}t^{½} + (0.1\mathrm{T}/\pi)^{½}(0.023RH + 0.0184\mathrm{NO}_{2(g)} + 0.0122\mathrm{O}_{3(g)})t \quad (11)$$

$$\Delta E = 0.4383+0.5979(Gt^{0.4})^{½} + (0.1\mathrm{T}/\pi)^{½}(0.0354RH + 0.0184\mathrm{SO}_{2(g)} + 0.0195\mathrm{O}_{3(g)})t^{0.65} \quad (12)$$

where $\Delta x$ is depth in microns, $\Delta E$ is the color change, and $G$ is solar irradiance expressed as $Wm^{-2}$. Here, these damage rates were determined for the first year, so t was set to unity.

### Paint

Whitehouse[31] suggests that a 30-ppb increase in ozone produces a 3% increase in the erosion rate of oil-based house paint and a 5% increase in the rate of damage to acrylic coil coatings. The ICP program offers equations[21] for the damage in terms of ASTM rankings, where 10 is a fresh sample and 1 is completely degraded. These rankings are an assessment of damage, but to avoid complexity here, we simply refer to them as time-dependent damage $D$ (i.e., 10-ASTM) for coil coated (cc) galvanized steel with alkyd melamine as:

$$D = (0.0084\mathrm{SO}_{2(g)} + 0.015RH + f_{cc} + 0.82Rn)t^{0.43} \quad (13)$$

where $f_{cc}$ is 0.04($T$-10) when $T < 10^oC$ and –0.064($T$-10) at $T > 10^oC$ and steel panels (sp) with alkyd as:

$$D = (0.033\mathrm{SO}_{2(g)} + 0.013RH + f_{sp} + 1.3Rn)t^{0.41} \quad (14)$$

where $f_{sp}$ is 0.015($T$-11) when $T < 11^oC$ and –0.15($T$-11) at $T > 11^oC$.

### Rubber

Damage to rubber by ozone is readily recognized, and so was well known even to the discoverer of photochemical air pollution, Haagen-Smit[32]. Holland et al.[7] suggest that the reciprocal time for the first appearance of cracks ($1/t_c$) can be described as:

$$1/t_c = r_e + k_{O3}\ O_3 \quad (15)$$

where $r_e$ is an environmental factor that includes the effect of imposed stress, sunlight, and temperature. We have not been able to use these as variables in the calculations here, so it essentially represents ozone attack at temperatures typical of the laboratory and in shaded conditions. The parameter $k_{O3}$ is a rate constant and $O_3$ is the ozone concentration in ppb. The values $r_e$ and $k_{O3}$ are both dependent on the type of rubber, and whether the crack formation is considered at edges or not. The values of $r_e$ and $k_{O3}$ adopted here are $19.5 \times 10^{-4}\ h^{-1}$ and $0.58 \times 10^{-4}\ h^{-1}$ for the edges, and $17.1 \times 10^{-4}\ h^{-1}$ and $0.51 \times 10^{-4}\ h^{-1}$ for the middle of rubber strips, respectively[7].

### RESULTS AND DISCUSSION

Many of the regression equations for metals have nonlinear parameters and although these represent the dataset from which they derive, any extrapolation has to be done with caution. Additionally, the nonlinear nature of these functions with time can be problematic. We have used yearly average data, but some of the equations refer to monthly averages. However, we explored the impact of this and saw that it is a particular problem at high pollutant concentrations because it is these that typically have noninteger exponents. It emerged in trial runs that taking the yearly average rather than the monthly average at high pollutant concentrations overestimated damage (by as much as 30%). At more modest pollutant concentrations, the annual average and monthly average values agree reasonably well. This bias would be serious if the corrosion peaks suggested for the 20th century (Fig. 2) were thus an artefact, but these are large enough for this not to be the case.

The recession rate for limestone in London (Fig. 2a, dotted line) shows a return to a dominance of the natural karst effect (carbon dioxide weathering) as noted in a previous work[12]. The same is true of Prague, but a lower pollution impact in the 20th century makes this transition less obvious (Fig. 2b).

The functions for aluminum are not linear with respect to time in the ICP equations, so the values plotted are essentially the initial annual corrosion rates. This is also true of the ICP functions for zinc and copper. Aluminum shows a clear decline in the rate of mass loss (Fig. 2a,b) and while this is again more obvious for the last half of the 20th century in London than in Prague, it is evident for both cities and the principal driver is sulfur dioxide. The decrease continues through the 21st century in London, but damage remains more static in the projection for a future Prague, where increases in temperature maintain the corrosion rates.

The picture for zinc (Fig. 2c,d) is somewhat similar to that for aluminum, and there is a reasonable agreement between the functions from the ICP and MULTI-ASSESS projects. The rate of damage tends to decline through the early part of the record because it is sensitive to sulfur dioxide. However, the mass loss predicted for Prague from MULTI-ASSESS shows a slight increase through the 21st century (Fig. 2d, square points) due to the effect of increasing ozone concentration (see Eq. 5). The ICP data appear in Fig. 2c–f as a shaded area, where the upper bound is for unsheltered samples and the lower bound is for sheltered exposures.

The mass loss for copper in London (Fig. 2e) repeats the pattern for zinc, although the NMEP equation gives rather higher values. However, copper has a complex corrosion process, which can yield both weight loss and weight gain through patination[21,33,34]. Early in the record, high corrosion rates are driven by rainfall acidity, but the predicted annual mass loss of copper in Prague (Fig. 2f) shows a slight increase through the 21st century, as the damage is influenced by the dependence of copper corrosion on ozone, which is taken to rise at this site (see Eqs. 6–8).

Plastics show a slight increase in damage rates or rather stable situations across the 21st century (Fig. 2g,h). This is often driven by the influence of solar radiation and temperature increases. HADCM3a2 suggests that irradiation in London will increase about 10% and Prague about 8%. There are problems with such estimates, as the effect of solar radiation on plastics and paint will also be highly dependent on the wavelength, and the transmission through the atmosphere can be dependent on pollution. The probable long-term change in UV at cities in reasonably high latitudes may well be a function of depletion of polar stratospheric ozone. The functions from the ICP project for coatings: (1) steel panels with alkyd and (2) coil coated galvanized steel with alkyd melamine (shown as the upper and lower bounds of the dark shaded area of Fig. 2g,h), give the familiar form of decline, resulting due to air quality improvements of the late 20th century. The amelioration is driven by the dependence of damage on sulfur dioxide concentrations inherent in Eqs. 13 and 14. The relationship for paint damage from Whitehouse[31] (light shaded area) is a function of ozone; hence, the notable increase in rate of deterioration predicted for Prague where ozone was set to rise (Fig. 2h).

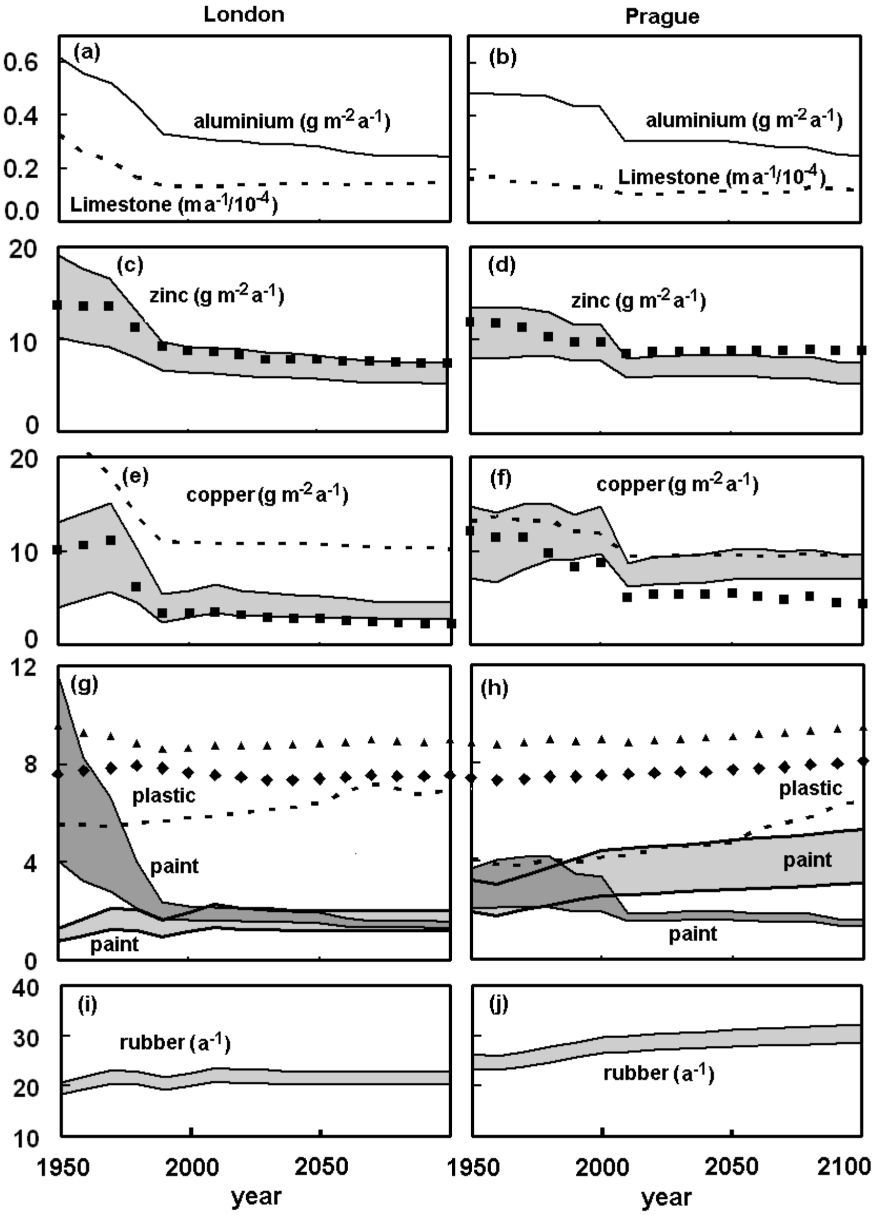

**FIGURE 2.** (a,b) Initial yearly rate of mass loss for exposed aluminum in London and Prague along with the recession to limestone. (c,d) Initial yearly rate of mass loss for zinc in London and Prague. The shaded area marks the lower bound (sheltered) and the upper bound (the exposed) metal from the ICP equation. The points mark the values from the MULTI-ASSESS equation. (e,f) Initial yearly rate of mass loss for copper in London and Prague. The shaded area marks the lower bound (sheltered) and the upper bound (the exposed) metal from the ICP equation. The points mark the values from the MULTI-ASSESS equation and the dotted line is from the NMEP function. (g,h) Rate of deterioration of plastic (units in text) in London and Prague in terms of changes in IR absorption in polyethylene (dotted line), damage depth in polyurethane (diamonds), and color change in polyester (triangles). Damage to paints is shown as ICP estimates for damage to steel panels with alkyd and coil coated galvanized steel with alkyd melamine, in ASTM rankings, as the upper and lower bounds of the dark shaded area and second, in light shading, Whitehouse's[31] estimates of the % increase in erosion rate of acrylic coil coating (upper bound) and oil-based house paint (lower bound). (i,j) Rate of damage to rubber in London and Prague[14,15], where the lower bound is the middle of the rubber and the upper bound the edges.

The results for rubber are rather similar to those from the relationship for paint damage from Whitehouse[31]. The results (Fig. 2i,j) suggest increased degradation of natural rubber in Prague because of rising ozone concentrations. Natural rubber finds few uses because of its sensitivity to ozone. Typically, rubber, both natural and artificial, is protected with antiozonants. Although these are effective, rising ozone concentrations could reduce the useful life of these additives[7].

### CONCLUSIONS

The picture we gain from this study is reassuring in its suggestion that for many of the important materials, such as stone, aluminum, zinc, and copper, the future looks better than the recent past. Improvements in air quality seem the most relevant driver for this amelioration. Although climate is set to change over the 21st century, these changes do not alter the picture. On the other hand, polymeric materials, plastic, paint, and rubber could well show slightly increased rates of degradation. This is hardly unexpected, and although the damage or dose-response functions for these materials are not as widely applied, the sensitivity to increased temperature, sunlight (as global radiation), and ozone makes future environmental threat to these materials worthy of further research. The management and maintenance of buildings may have to recognize a changed climate and its importance in damage to the urban fabric.

### ACKNOWLEDGMENTS

This paper was a development from work done with the EU-funded project "*NOAH's ARK": Global Climate Change Impact on Built Heritage and Cultural Landscapes* (http://noahsark.isac.cnr.it/), which projected climate change effects into the future. We greatly benefited from our colleagues at NOAH's ARK who taught us so much that contributed to the current work. In addition, we owe our very special thanks to Ian Harris of the Climate Research Unit who was always there when we needed climate predictions.
